# Cardiac dysfunction in survivors of sepsis: a scoping review

**DOI:** 10.1136/openhrt-2023-002454

**Published:** 2023-12-08

**Authors:** Kevin Garrity, Samantha Gaw, Alice Blewitt, Paul Canon, Philip McCall, Joanne McPeake

**Affiliations:** 1 University of Glasgow, Glasgow, UK; 2 NHS Greater Glasgow and Clyde, Glasgow, UK; 3 NHS Ayrshire and Arran, Kilmarnock, UK; 4 NHS Golden Jubilee, Glasgow, UK; 5 University of Cambridge, Cambridge, UK

**Keywords:** heart failure, risk factors, biomarkers, outcome assessment, health care, cardiac imaging techniques

## Abstract

**Background:**

Sepsis is associated with an increased risk of adverse cardiovascular events in a magnitude comparable to other major cardiovascular risk factors. Sepsis is one of the most common reasons for intensive care admission and survivors often have significant functional limitations following discharge. However, it is not clear to what extent chronic cardiovascular dysfunction might mediate these functional impairments, or how we might screen and manage these patients at risk of chronic cardiovascular disease. We conducted a scoping review to map existing evidence and identify research gaps relating to cardiovascular dysfunction following sepsis.

**Methods:**

We conducted a systematic search of MEDLINE, Embase and CINAHL databases using a concept, context, population (CoCoPop) framework. Studies examining cardiovascular outcomes or symptoms following an episode of sepsis in adults were included. Data were mapped based on the population assessed, cardiovascular outcomes examined, inclusion of objective measures of cardiac dysfunction such as biomarkers or cardiovascular imaging, or whether cardiovascular symptoms or patient-reported functional outcomes measures were recorded.

**Results:**

We identified 11 210 articles of which 70 were eligible for full text review and 28 were included in final analysis. Across our dataset, a wide range of incident cardiovascular outcomes were reported in the literature including incidence of congestive heart failure (13/28), arrhythmia (6/28), myocardial infarction (24/28) or cardiovascular death or all-cause mortality (20/28). Only 39% (11/28) of articles reported objective measures of cardiovascular function and only one article related cardiovascular function to functional impairment via patient-reported outcome measures.

**Conclusion:**

There are significant gaps in our understanding of cardiac dysfunction following sepsis . While the research highlights the strong association of sepsis with a variety of adverse cardiovascular outcomes, further prospective work is required to understand the mechanisms that mediate this phenomenon and how we can best identify and manage patients at risk.

**WHAT IS ALREADY KNOWN ON THIS TOPIC**

- There is an increasing evidence that admission with sepsis—one of the most common reasons for intensive care or hospital admission—is associated with a long-term risk of adverse cardiovascular events. The extent to which current evidence explores mechanisms that might mediate adverse cardiovascular events following sepsis, or whether chronic cardiovascular dysfunction is implicated in impaired recovery, is unclear.

**WHAT THIS STUDY ADDS**

- There is a burgeoning interest in sepsis as a non-traditional cardiovascular risk factor through its association with adverse cardiovascular events following admission. However, significant gaps in the literature remain that would help in understanding mechanisms by which these events are mediated, what patient groups are most at risk and whether this risk might be modified.

**HOW THIS STUDY MIGHT AFFECT RESEARCH, PRACTICE OR POLICY**

- We urgently need prospective data sets that allow us to understand causative mechanisms implicated in cardiovascular dysfunction following sepsis, which patients are most at risk of cardiovascular events and whether this apparent cardiovascular risk might be reduced by established preventative or disease-modifying therapies.

## Background

Sepsis is one of the most common reasons for admission to intensive care and one of the leading causes of mortality worldwide.^1–3^ The impact of critical illnesses such as sepsis are profound, with up to two-thirds of intensive care survivors experiencing either cognitive, psychological or functional impairments now collectively known as postintensive care syndrome.^4–6^ There is an increasing recognition that, like the index episode of sepsis itself, these lingering impairments are likely complex and heterogeneous in nature and a result in interactions between patient characteristics, specific pathophysiological processes and factors related to treatments.^7^ Nonetheless, mechanisms by which these functional impairments are mediated following admission are unclear.

One potential mechanism of functional impairment may be chronic cardiac dysfunction.^8–10^ In recent years, several large cohort studies and a systematic review have demonstrated that admission with sepsis is associated with a higher incidence of cardiovascular events such as myocardial infarction and heart failure, with rates that are comparable to other established cardiovascular risk factors such as hypertension or dyslipidaemia.^8 9 11–13^

Given the association of sepsis with an increased risk of adverse cardiovascular outcomes, it is logical then that chronic cardiovascular dysfunction may be common following critical illness and may play a role in the functional impairments observed following admission. Furthermore, the association of sepsis admission and adverse cardiovascular events potentially points towards a plausible mechanism by which critical illness such as sepsis, might accelerate or even mediate chronic cardiovascular disease. Dysregulated inflammation that is the basis of contemporary definitions of sepsis may provide a logical pathway by which cardiovascular events are accelerated or might even occur de novo.^14 15^ However, while the literature to date has demonstrated an association with sepsis and adverse risk of cardiovascular outcomes, evidence is lacking regarding mechanisms of chronic cardiac dysfunction following acute illness, its relationship to functional impairments seen in survivors of sepsis and how we might screen for and manage at risk patient groups. We were keen to identify available types of evidence, clarify key concepts and examine how research has been conducted in the field to date. As such, we conducted a scoping review on the topic.

## Methods

The scoping review aimed to answer the following interrelated questions:

- To what extent does current literature explore cardiovascular function or dysfunction following an episode of sepsis?
- What research methodologies have been employed to explore the long-term cardiovascular effects of hospital admission with sepsis?
- To what extent does this literature explore the role cardiovascular function might play in functional impairment following admission with sepsis?
- Does this literature explore any mechanisms by which cardiovascular dysfunction might cause long-term functional impairment (eg, objective evidence of ischaemia or heart failure)?

### Protocol and registration

A scoping review strategy was based on an a priori suspicion that beyond incident outcomes, there would be few prospective studies examining this topic that would allow for synthesis and meta-analysis of data. The review was conducted in alignment with the Joanna Briggs Institute (JBI) guidance and reported in accordance with Prefered Reporting Items for Systematic Reviews and Meta-Analyses Scoping Review (PRISMA-ScR) standards.^16^ The study protocol and search strategy were registered on Open Science Framework (https://doi.org/10.17605/OSF.IO/QPSY5) and checklist is included in online supplemental files.

10.1136/openhrt-2023-002454.supp1 Supplementary data

### Search strategy, sources and eligibility criteria

Using an initial focused search, we identified key papers on the topic to devise a comprehensive search strategy in conjunction with an experienced librarian. We subsequently searched MEDLINE, Embase and CINAHL using the Concept, Context, Population (CoCoPop) framework as outlined by the JBI for use in scoping reviews.^17^

The search strategy (online supplemental appendix I), including text words and subject headings, was adapted for each database. The reference list of all included sources of evidence was screened for additional eligible articles.

#### Participants

This scoping review focused on adult survivors of sepsis and included any literature which considered outcomes for adult patients >18 years old, regardless of comorbidity or frailty.

#### Concept

The search strategy was aimed at identifying literature with the terms ‘heart failure’ or ‘cardiovascular outcomes’ in the title or abstract, focusing on the concept of cardiac dysfunction following an episode of sepsis. Papers that focused on acute haemodynamic instability during an episode of sepsis (eg, septic shock or acute arrhythmia) were not included unless they contained data pertaining to long-term cardiovascular outcomes following discharge from hospital.

#### Context

We focused on sepsis survivorship. The terms ‘sepsis’, ‘bacteraemia’ and other related terms were used to identify relevant literature for potential inclusion. The ‘sepsis 3’ definitions are widely recognised by the sepsis and critical care research communities and define sepsis as life-threatening organ dysfunction caused by a dysregulated host response to infection. Data in this scoping review used this definition for inclusion of articles in this review.^14^

### Sources and selection of evidence

Prospective and retrospective observational or cohort studies were included. In addition, the review considered both experimental and quasi-experimental designs including randomised controlled trials, non-randomised controlled trials, before and after studies and interrupted time-series studies. The review also considered descriptive observational study designs including case series, individual case reports and descriptive cross-sectional studies for inclusion. Conference proceedings, conference abstracts or book chapters were not reviewed to avoid duplication that may have occurred in published studies.

Following the search, all identified citations were collated and uploaded into the Rayyan platform and duplicates removed. A pilot test ensuring reviewer agreement (>75%) was conducted prior to screening results. Subsequently, titles and abstracts were then screened by two or more independent reviewers for assessment against the inclusion criteria for the review using Rayyan software. Any disagreements that arose between reviewers at each stage were resolved through discussion, or with (an) additional reviewer(s). The results of the search and the study inclusion process were reported using the (PRISMA-ScR) flow diagram.^16^

### Data extraction, analysis and presentation

Data were extracted from by two or more independent reviewers using the data extraction tool (online supplemental appendix II). The data extracted included specific details about the participants, concept, context, study methods and key findings relevant to the review questions. We identified patients who had sepsis as defined by sepsis 3 criteria.^14^ Where patients were not explicitly identified as having sepsis by the authors, we included the paper only if sepsis was present in the majority of participants (>50%).

The data extraction tool was developed a priori and was modified and revised as necessary during the process of extracting data from each included evidence source. Any disagreements that arose between the reviewers were resolved through discussion, or with (an) additional reviewer(s).

The literature was collated and mapped according to the review questions. Literature was tabulated by author, date, population, context, relevant clinical outcomes and by methods that gave mechanistic insights into long-term cardiovascular outcomes (ie, cardiac biomarkers, electrocardiographic findings, cardiac imaging). In addition, we mapped articles according to whether they documented cardiovascular symptoms or patient-reported outcome measures. Authors subsequently identified gaps in the literature with a view to formulating questions for further research.

In keeping with JBI guidance on conducting scoping reviews, we did not conduct meta-analysis as this would have required assessment for bias and compromised our ability to map gaps in the literature through exclusion of articles.^18^

## Results

### Summary

**Figure 1:**
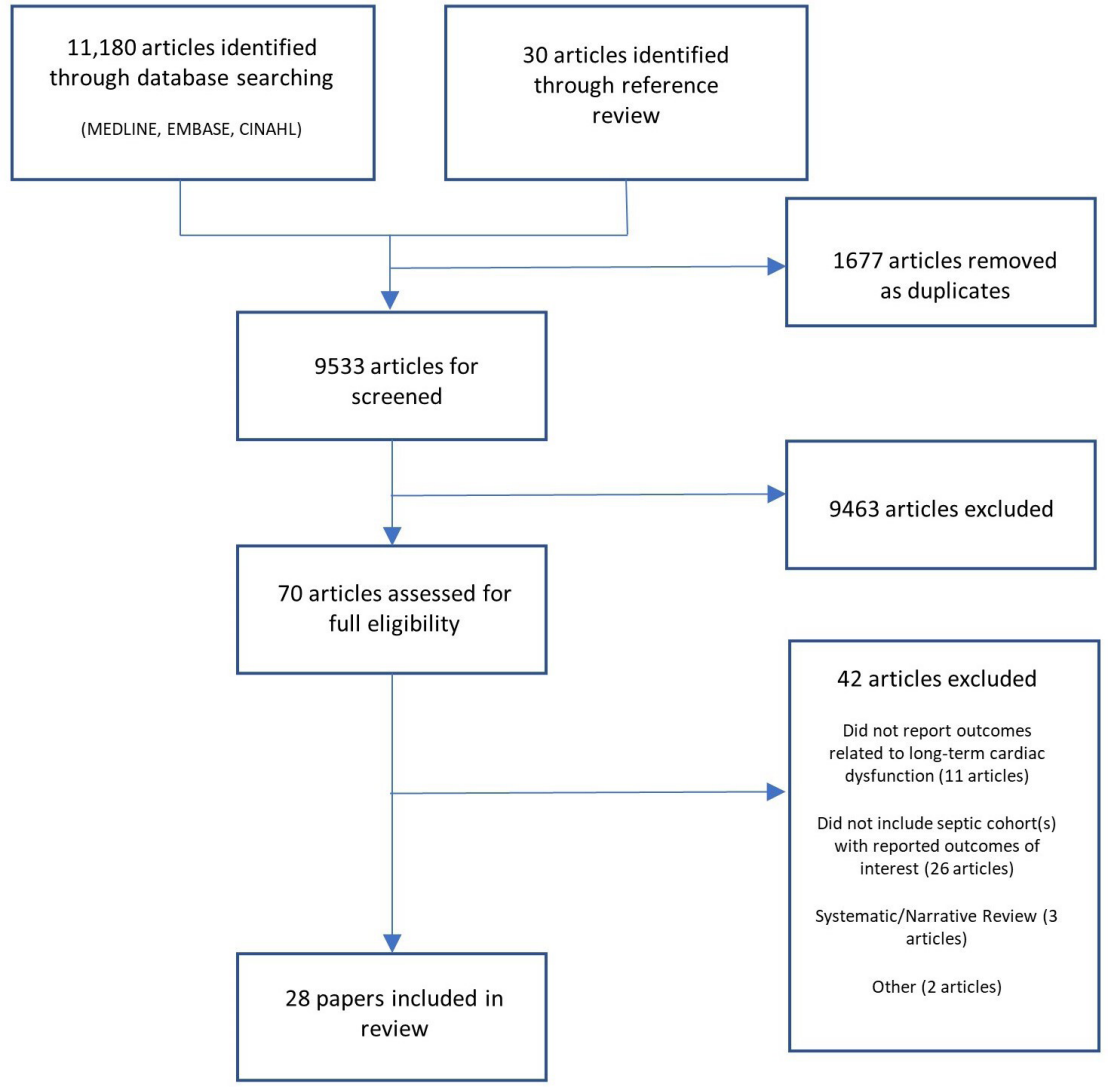
Flow chart of included studies.^16^

The study selection process is outlined in figure 1. In total, 11 210 individual articles were identified for review. From these articles, 9533 were eligible for screening after removal of duplicates and subsequently 70 articles were eligible for full text review. Forty-two articles were removed from the final data set. Of these papers, 26 articles were removed because they did not contain septic cohorts with reported outcomes of interest, 11 articles were removed because they did not report outcomes related to long-term cardiac dysfunction, 1 article was removed as it was a prospective study protocol with no results as yet and 3 articles were narrative or systematic reviews. One further article was removed as it was a pathological study with no focus on long-term cardiovascular dysfunction.

### Characteristics of studies

Of the 28 articles included (table 1), all discussed long-term outcomes related to cardiovascular dysfunction in patients who had sepsis. Articles covered a 17-year period from 2005 to 2023 and studied patient populations in a variety of geographical locations including, North America (19/28), Europe (4/28) and Asia (5/28). Articles focused on a wide range of follow-up periods from 30 days up to 10 years. All included articles studied adult patients, of which two focused on particular subgroups of patients vulnerable to cardiovascular disease (patients with chronic kidney disease and patients with end-stage renal failure commencing dialysis).^19 20^ In total, 82% (23/28) of articles explicitly described patient cohorts who had sepsis; however, 18% (5/28) were articles in which the primary concept was pneumonia, but explicitly reported outcomes related to cardiovascular dysfunction for septic patients.

**Table 1:** Summary of articles included in scoping review^8 10 12 13 19–28 37–50^

First author	Year	Country	Context	Concept	Population	Comparator cohort	Methodology	CV outcomes studied	Mechanisms of CV dysfunction assessed
Adamuz^37^	2014	Spain	Pneumonia	Predictor of 1- year mortality	Adults	Alive versus dead	Prospective Cohort Study	MI, CV deaths	–
Aldás^38^	2020	Spain	Pneumonia	MACE	Adults	MACE versus no MACE	Prospective Cohort Study	Heart failure, arrhythmia, MI, all-cause mortality	–
Alnabelsi^25^	2020	USA	Sepsis	MACE	Adults	CTCA and reduced LVEF versus low CTCA and normal LVEF	Retrospective Cohort Study	MI, CV deaths	Imaging, biomarkers
Angriman^39^	2023	Canada	Sepsis	Risk factors associated with MACE	Adults	RAASi versus no RAASi	Retrospective Cohort Study	MI, all-cause mortality	Biomarkers
Angriman^21^	2023	Canada	Sepsis	RAASi and MACE	Adults	MACE versus no MACE	Retrospective Cohort Study	MI, all-cause mortality	RAASi prescribing
Angriman^12^	2022	Canada	Sepsis	MACE	Adults	Sepsis versus hospitalised non-sepsis	Retrospective Cohort Study	MI, CV deaths	–
Beesley^26^	2021	USA	Sepsis	MACE	Adults	Normal versus abnormal GLS	Retrospective Cohort Study	Heart failure, MI, all-cause mortality	Imaging, biomarkers
Chang^40^	2015	USA	Sepsis	Hospital readmissions and diagnoses	Adults	Sepsis versus AMI versus CHF	Retrospective Cohort Study	MI, CV admissions	–
Corrales-Medina^10^	2009	USA	Pneumonia	Acute coronary syndrome	Adults	Pneumonia versus hospitalised control	Retrospective Cohort Study	MI	–
Corrales-Medina^13^	2015	USA	Pneumonia	MACE and mortality	Adults	Pneumonia versus hospitalised control	Retrospective Cohort Study	MI, CV death	–
De Geer^41^	2018	Scandinavia	Sepsis	CV mortality	Adults	Sepsis versus hospitalised non-sepsis	Retrospective Cohort Study	CV death	–
Gadre^42^	2019	USA	Sepsis	Hospital readmissions	Adults	30-day readmission versus no 30-day readmission	Retrospective Cohort Study	CV admissions	–
Garcia^43^	2021	USA	Sepsis	MACE, mortality	Adults	Normal versus abnormal troponin values	Retrospective Cohort Study	Heart failure, arrhythmia, MI, all-cause mortality	Biomarkers
Gupta^22^	2018	USA	Sepsis	Coronary artery disease and mortality	Adults	CAC absent versus CAC present	Prospective Cohort Study	MI, CV death	Imaging
Gupta^27^	2020	USA	Sepsis	Statin prescribing following admission with sepsis	Adults	Statin indication versus no statin indication	Retrospective Cohort Study	MI, heart failure, CV death	Statin prescribing
Ishani^44^	2005	USA	Sepsis	MACE	Adults with end stage renal failure	Sepsis versus population control	Retrospective Cohort Study	Heart failure, MI, all-cause mortality	–
Jafarzadeh^19^	2016	USA	Sepsis	MACE	Adults	Sepsis versus hospitalised non-sepsis	Retrospective Cohort Study	MI	–
Jentzer^45^	2023	USA	Sepsis	MACE	Adults	Sepsis versus hospitalised non-sepsis	Retrospective Cohort Study	CV death, heart failure, ischaemic heart disease, MI, arrhythmia, all-cause mortality	–
Lai^46^	2018	Taiwan	Sepsis	MACE	Adults	Sepsis versus hospitalised non-sepsis versus population control	Retrospective Cohort Study	MI	–
Menéndez^24^	2019	Spain	Pneumonia	MACE	Adults	Early versus late CV events	Retrospective Cohort Study	Heart failure, arrythmia, MI, all cause-mortality	Biomarkers
Ou^47^	2016	Taiwan	Sepsis	MACE	Adults	Sepsis versus hospitalised non-sepsis versus population control	Retrospective Cohort Study	Heart failure, MI, arrhythmia, all-cause mortality	–
Ou^23^	2021	Taiwan	Sepsis	MACE, RAASi prescribing	Adults	RAASi versus no RAASi	Retrospective Cohort Study	Heart failure, MI, all-cause mortality	RAASi prescribing
Prescott^48^	2017	USA	Sepsis	Hospital readmissions	Adults	<65 years versus ≥65 years	Retrospective Cohort Study	CV admissions	–
Shih^20^	2007	Taiwan	Sepsis	MACE	Adults with CKD	Sepsis versus hospitalised non-sepsis	Retrospective Cohort Study	Heart failure, MI, all-cause mortality	–
Vallabhajosyula^28^	2018	USA	Sepsis	Mortality, heart failure	Adults	Sepsis LV dysfunction versus sepsis no-LV dysfunction	Retrospective Cohort Study	Heart failure, all-cause mortality	Imaging, biomarkers
Walkey^49^	2014	USA	Sepsis	Mortality	Adults	No AF versus new-onset AF versus prior AF	Retrospective Cohort Study	Heart failure, arrhythmia, MI	ECG analysis
Wu^50^	2019	Taiwan	Sepsis	MACE	Adults	CVE versus no CVE	Retrospective Cohort Study	MI, all-cause mortality	–
Yende^30^	2014	USA	Sepsis	MACE, mortality	Adults	Sepsis (ICU and non-ICU) versus hospitalised sepsis (ICU and non-ICU) versus population control	Retrospective Cohort Study	MI	–

AF, atrial fibrillation; AMI, acute myocardial infarction; CHF, congestive heart failure; CKD, chronic kidney disease; CTCA, computed tomography coronary angiogram; CV, cardiovascular; CVE, cardiovascular event; GLS, global longitudinal strain; ICU, intensive care unit; LV, left ventricular; LVEF, left ventricular ejection fraction; MACE, major adverse cardiovascular event; MI, myocardial infarction; RAASi, renin–angiotensin–aldosterone inhibitor.

### Research methodology

All included articles were either prospective (4/28) or retrospective (24/28) cohort studies. Although there was one systematic review and meta-analysis, we excluded this from our final analysis on the basis that it contained duplicate data from many of the articles presented. There was a wide range in methodology, with many studies comparing sepsis cohorts with hospitalised non sepsis or population controls. Some studies used findings related to cardiovascular imaging (4/28) or biomarkers (3/28) to stratify patients into groups of varying risk of cardiovascular events. We could find no randomised controlled trials investigating interventions related to reducing risk of adverse cardiovascular events following sepsis, although three retrospective observational studies focused on cohorts of patients prescribed statins or renin–angiotensin–aldosterone (RAAS) inhibitors, drugs commonly used in management or prevention of cardiovascular disease, versus those who were not.^21–23^

### Cardiovascular outcomes

**Figure 2:**
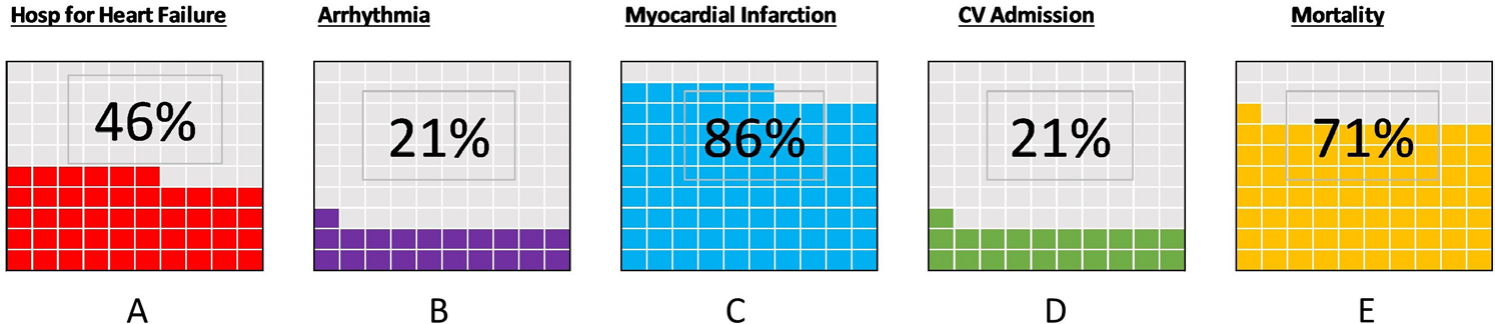
Long-term cardiovascular outcomes in sepsis survivors, and frequency in which they are reported in the literature. (A) Hospitalisation with heart failure, (B) arrhythmia, (C) myocardial Infarction, (D) cardiovascular admissions, (E) mortality.

The included articles reported a variety of incident outcomes related to cardiovascular function following sepsis (figure 2). Eighty-six per cent of articles (24/28) reported incidence of myocardial infarction or acute coronary syndromes and 46% (13/28) reported admissions or presentations with congestive heart failure. Seventy-one per cent (20/28) of articles reported either all-cause or cardiovascular mortality and 21% (6/28) examined cardiovascular admissions to hospital. Indeed, two articles focused explicitly on hospital admissions and diagnoses following sepsis.

Other commonly reported outcomes related to long-term cardiovascular dysfunction included incidence of atrial fibrillation or other dysrhythmia, with 21% (6/28) of articles described in patients with sepsis at follow-up.

Despite a wealth of data describing incident outcomes such as congestive heart failure, myocardial infarction or cardiovascular admissions, we could find no papers which reported any objective cardiovascular symptoms and only one study referenced patient-reported measures of functional impairment.^10^

### Mechanisms of cardiovascular dysfunction following sepsis

**Figure 3:**
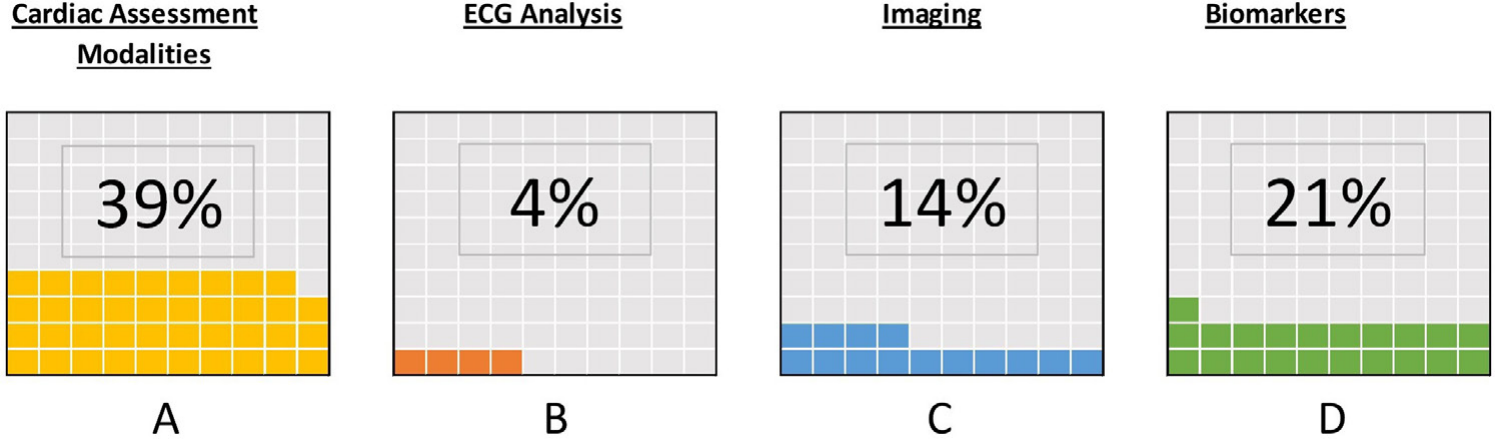
Modalities of cardiovascular assessment in identified literature. (A) Proportion of papers that include any modalities of cardiac assessment and relate them to long-term cardiovascular outcomes. (B–D) Proportion of papers in the literature that include outcomes related to ECG findings (B), findings on imaging (C) or biomarker analysis (D).

Thirty-nine per cent (11/28) of articles reported variables or outcomes related to cardiac assessment modalities, which could give mechanistic insights into the incident outcomes reported (figure 3). There was wide variation in methodology employed and modalities of cardiac assessment used.

Twenty-one per cent (6/28) of studies reported cardiac or inflammatory biomarkers as a variable of interest. Five of the included articles used troponin during index illness as a variable for inclusion in data analysis or to risk-stratify patients into separate cohorts for study. Only one study analysed other cardiac or inflammatory biomarkers (eg, N-terminal-pro B-type natriuretic peptide (NT-proBNP) or acute phase inflammatory biomarkers). This particular study was the only article which described serial biomarkers that were sampled beyond admission.^24^

Fourteen per cent (4/28) of articles related findings on imaging to cardiovascular outcomes.^25–28^ All of these articles focused on imaging findings during index illness and employed various of modalities (CT-coronary angiography or echocardiography) to group patients into hypothetical groups of cardiovascular risk based on their imaging findings. There were no studies that used cardiac imaging undertaken during patient recovery and follow-up periods.

One study stratified patients based on the presence of atrial fibrillation on ECG and related this to long-term risk of cardiovascular events.

While not objective assessments of cardiovascular dysfunction, two studies explored the role of RAAS inhibition following admission with sepsis and found a reduction in risk of adverse cardiovascular events using retrospective cohort analysis that employed propensity scoring methodologies.^21 23^ We could find no prospective randomised controlled trials that explored the effect of preventative or disease-modifying therapies on the risk of adverse cardiovascular events.

## Discussion

**Figure 4:**
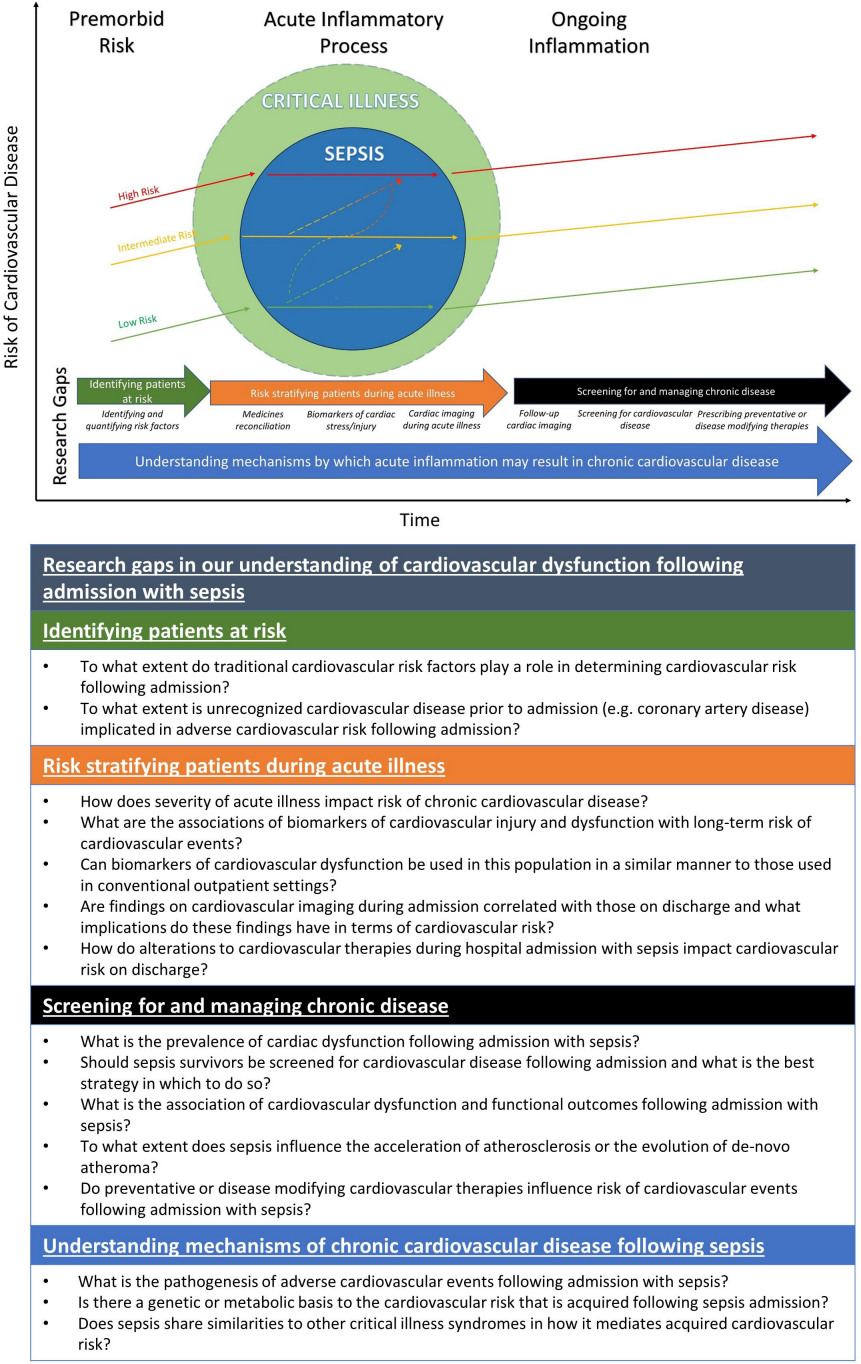
Understanding cardiovascular disease following sepsis and other critical illness and gaps in the current literature. A hypothetical model of cardiovascular dysfunction acquired as a consequence of sepsis. There are research gaps regarding how we might identify and quantify risk for different patient sub-groups; how we can identify patients most at risk during their hospital or ICU stay; how we optimally screen for and manage cardiovascular disease following admission; and in understanding fundamental mechanisms by which admission with sepsis mediates chronic cardiovascular disease.

We have mapped the literature pertaining to cardiovascular dysfunction following hospital admission with sepsis, the research methodologies employed and the extent to which the literature employs objective methods of cardiovascular assessment to explore this topic. This review demonstrates that while there is an established body of evidence to suggest that sepsis is associated with a long-term risk of cardiovascular events, there are gaps in our understanding as to why this occurs. Only 5/28 included studies (14%) were prospective in nature and only 11/28 (39%) included objective measurements of cardiac assessment, cardiac injury or functional outcomes following admission. While basic biological mechanistic research is out with the remit of this review, this scoping review demonstrates a lack of articles highlighting broad clinical mechanisms by which these events could be mediated and that there are several gaps in the literature for which there is a need to explore with further research (figure 4). We propose a hypothetical model of the risk of cardiovascular disease acquired as a consequence of sepsis and propose a range of research questions that require exploration.

First, despite a body of evidence suggesting that cardiac dysfunction following admission with sepsis is prevalent, the approach towards identifying at-risk groups on admission is less clear. Sepsis is one of the most common reasons for hospital and intensive care admission.^1–3^ As such, this population represents a huge cohort of patients at risk of unrecognised cardiovascular disease that may benefit from established preventative or disease-modifying therapies. Our scoping review demonstrates a clear recognition of the issue in the literature; however, there is clearly a varied approach to collecting data pertaining to adverse cardiovascular outcomes and identifying which patients may be at risk. All of the studies in this review are retrospective and observational in nature, using administrative datasets with ICD codes to record variables and outcomes. There can be variability in recording these outcomes and as such they may be vulnerable to confounding. Articles that attempted to further risk stratify patients further by using objective mechanistic data such as imaging or biomarkers were often retrospective or single centre studies and used differing methodologies that have variable availability in clinical practice. Thus, it remains unclear which objective methods of cardiovascular assessment may be useful for these purposes and if so, for which patient groups. We believe further study is required to delineate what patients are most at risk of cardiovascular events following admission with sepsis. Furthermore, we require new prospective data sets exploring how we might further stratify survivors using established modalities of cardiovascular assessment to identify patients at risk of adverse outcomes following discharge from critical care and hospital settings.

Second, the underlying mechanisms that mediate postsepsis cardiovascular events remain unclear. As eluded to above, less than half of articles included any objective assessment of cardiovascular dysfunction and consequently, there is a paucity of data exploring potential mechanisms for adverse outcomes. Only 21% of articles explored relationships with cardiac biomarkers and 14% examined imaging findings. Despite a focus on long-term cardiovascular outcomes, we could only find one study that prospectively collected mechanistic data beyond discharge—in this case, cardiac and inflammatory biomarkers—and explored their relationship with adverse cardiovascular events.^24^ Consequently, we conclude that the mechanisms underlying the acquired cardiovascular risk related to sepsis require further exploration. For example, there is little evidence examining whether sepsis has perhaps accelerated pre-existing chronic cardiovascular disease or whether it has occurred de novo. It remains unclear as to what extent this phenomenon is driven by atherosclerotic disease or whether myocardial inflammation might play a role. Studies that help characterise these underlying mechanisms—for example, by prospectively collecting cardiac imaging or biomarkers—in the sepsis survivor population are required to investigate whether proposed clinical mechanisms can be confirmed or refuted.

Third, the prevalence of chronic cardiac disease and how this affects survivors of sepsis on a day-to day-basis is unclear. Our scoping review identified just one article which linked admission with sepsis to cardiovascular outcomes and patient-reported functional outcomes measures or cardiovascular symptoms.^10^ Given the frequency of cardiovascular admissions following sepsis, it seems plausible that a significant proportion of sepsis survivors may be affected by chronic heart failure or other chronic cardiovascular disease. Future research should aim to address the long-term impact on survivors and how this relates to functional outcomes.

Fourth, given the potential prevalence of cardiovascular dysfunction in this large cohort of patients, it is tempting to consider the potential for cardiovascular therapies to modify risk. We could find no randomised controlled trials to prospectively explore this domain. Future prospective research should seek to study whether established cardiovascular therapies may lower risk of adverse cardiovascular events or indeed modify recovery trajectory. However, the research methodology employed is likely to be informed by improved understanding of the epidemiological and mechanistic factors related to these questions.

Finally, there is significant overlap between literature examining cardiovascular outcomes in specific infective processes (eg, pneumonia) and the sepsis syndrome. Our understanding of critical illness and how we define disease processes is changing, with the recognition that many classically labelled clinical syndromes may share pathophysiological mechanisms.^29^ Although there may be differences in terms of initial pathophysiological insults at the time of acute illness, the mechanisms that mediate the underlying increase in cardiovascular risk may be similar. Previous research has demonstrated that survivors of sepsis and pneumonia have increased circulating levels of proinflammatory cytokines following discharge.^8 24 30^ Similar inflammatory markers have been associated with occurrence of long-term adverse cardiovascular events, and critical illness in general is thought to induce immunometabolic changes associated with atherosclerotic disease.^15 31 32^ Indeed, some literature included in this scoping review also identified an increased risk of cardiovascular events in survivors of critical illness with ‘non-sepsis’ aetiologies.^9^ Further prospective research is required to understand this intersection between acute illness and chronic cardiovascular disease. As well as understanding how this risk is mediated in conditions such as sepsis, it is also important to consider whether it might occur as long-term sequelae of critical illness in general (figure 4).

There are a number of proposed mechanisms by which cardiovascular disease following critical illness might occur, including altered homoeostatic reflexes; alterations in immune signalling and lipid metabolism; and cellular level changes that all predispose to chronic cardiovascular disease.^15^ There has been burgeoning interest in the role inflammation may play in chronic heart failure and atherosclerotic disease and there have been exciting novel developments.^33 34^ For example, the Canakinumab Anti-Inflammatory Thrombosis Outcome Study (CANTOS) trial demonstrated that administration of anti IL-1β antibodies to patients postmyocardial infarction and treated in accordance with current guidelines were at reduced risk of adverse cardiovascular events.^35^ A subsequent subgroup analysis identified a significant reduction in hospitalisation or mortality from heart failure in patients who achieved low high-sensitivity C reactive protein on treatment.^36^ While this offers promise of novel, targeted treatments for heart failure, it too offers an insight into mechanisms by which persisting inflammation such as that following sepsis, might mediate cardiovascular dysfunction and contribute to functional impairment.

Strengths of this scoping review include the development of a robust search strategy developed in partnership with a librarian with extensive experience in the conduct of systematic review. Furthermore, we have rigorously adhered to JBI guidelines and Preferred Reporting Items for Systematic Reviews and Meta-analyses Scoping Review (PRISMA-ScR) standards.^16^ The pilot phase of our search strategy to ensure it was sensitive to collecting key papers that had previously been identified as highly relevant. Over 11 000 articles were screened by at least two independent reviewers who subsequently undertook detailed full text review of potential articles for inclusion. We deliberately focused on cardiovascular outcomes following an episode of sepsis and as such had an emphasis on the impact of cardiovascular disease on sepsis survivorship. There are some limitations to our review. Our focus was primarily on identifying literature pertaining to chronic cardiac dysfunction in the period following an episode of sepsis. As such, other outcomes such as acute stroke or venous thromboembolic events were not included. While these conditions have similar aetiologies, the assessment and long-term management of these conditions is different to that of cardiac dysfunction, consequently they were excluded from the review. Similarly, we ensured a focus on sepsis and not discrete infections (eg, urinary tract infection or cellulitis). Doing so would have led to an elaborate and inelegant search strategy that covered a wide range of infections of low severity that were not relevant to our outcomes of interest. We were also unable to use the scoping review to differentiate between literature around cardiovascular dysfunction following sepsis versus critical illness as a whole. We did not include non-english articles in our results, while our strategy identified some articles that were published in more than one language, it is possible we may have identified further articles on this topic by inclusion of other languages. However, attempting to include these articles would have likely introduced errors in translation and consequently may have confounded our data set.

To conclude, while there is an evolving body of literature examining cardiovascular dysfunction following sepsis, there is still a lack of data examining underlying mechanisms by which this occurs and how it might translate to chronic cardiovascular disease and functional impairment. The strategy by which we should identify at risk groups of patients or screen and manage chronic cardiovascular disease on discharge is unclear. Further prospective studies are required to explore these mechanisms and understand how future research and clinical interventions may mitigate this increased cardiovascular risk, with the aim of improving the trajectory of recovery for survivors of sepsis.

## Data Availability

Data are available in a public, open access repository.
