# Supplementary material for: Cardiac dysfunction in survivors of sepsis: a scoping review
Source: Open Heart. 2023 Dec 8;10(2):e002454. doi: 10.1136/openhrt-2023-002454 (PMC10711866; doi:10.1136/openhrt-2023-002454)
Supplement: Supplementary data [file openhrt-2023-002454supp001.pdf]

Supplementary Appendices

Appendix I: Search strategy

Medline Search Strategy

#	Query
1	Cardiovascular Diseases/
2	Heart Failure/
3	heart failure.mp.
4	cardi* failure.mp.
5	cardi* dysfunction.mp.
6	cardiogenic shock.mp.
7	cardiovascular outcomes.mp.
8	1 or 2 or 3 or 4 or 5 or 6 or 7
9	Sepsis/
10	Bacteremia/
11	bacter?emia.mp.
12	endotox?emia.mp.
13	Shock, Septic/

14	(Septic* or Seps*).mp. [mp=title, abstract, original title, name of substance word, subject heading word, floating sub-heading word, keyword heading word, organism supplementary concept word, protocol supplementary concept word, rare disease supplementary concept word, unique identifier, synonyms]
15	pneumonia.mp.
16	9 or 10 or 11 or 12 or 13 or 14 or 15
17	exp Adult/
18	Cohort Studies/
19	Retrospective Studies/
20	Prospective Studies/
21	Longitudinal Studies/
22	(follow-up or long-term or cohort or longitudinal or prospective or retrospective).mp. [mp=title, abstract, original title, name of substance word, subject heading word, floating sub-heading word, keyword heading word, organism supplementary concept word, protocol supplementary concept word, rare disease supplementary concept word, unique identifier, synonyms]
23	18 or 19 or 20 or 21 or 22
24	8 and 16 and 17 and 23
25	limit 24 to English language

Appendix II: Data extraction instrument

**Scoping Review Data Extraction Instrument**	
Scoping Review Title:	Cardiac Dysfunction in Survivors of Sepsis
Review Objective:	Map evidence regarding cardiac dysfunction following sepsis and examine how it might be implicated in functional impairment following intensive care
Review Questions(s):	• To what extent does current literature explore cardiovascular function or dysfunction *following* an episode of sepsis?<br>• What research methodologies have been employed to explore the long-term cardiovascular effects of hospital admission with sepsis?<br>• To what extent does this literature explore the role cardiovascular function might play in functional impairment following admission with sepsis?<br>• Does this literature explore any mechanisms by which cardiovascular dysfunction might cause long-term functional impairment (e.g. objective evidence of ischaemia or heart failure)
**Inclusion/Exclusion Criteria**	
Population	Adults
Concept	Cardiac Dysfunction during follow-up
Context	Admission with sepsis
**Evidence Source Details and Characteristics**	
Citation Details	

Country	
Context	
**Details/Results Extracted from Source of Evidence**	
Patient Reported Outcome Measures (e.g. performance status, functional questionnaire, NYHA)	
ECG findings reported?	
Cardiac Imaging Reported? (e.g. Echocardiographic Findings / CMR)	
Cardiac Biomarkers (e.g. NT-proBNP, Troponin)	
Incident CV outcomes reported (e.g. MI/Heart Failure Admission)	
